# Association between unhealthy snack food and beverage consumption and the nutritional status of Nepalese children and adolescents: A systematic review

**DOI:** 10.1371/journal.pone.0352341

**Published:** 2026-06-25

**Authors:** Rameshwor Parajuli, Wilna Oldewage-Theron, Surya Raj Niraula

**Affiliations:** 1 Central Department of Zoology, Tribhuvan University, Kirtipur, Nepal; 2 Department of Sustainable Food Systems and Development, Faculty of Natural and Agricultural Sciences, University of the Free State, Bloemfontein, South Africa; 3 Department of Nutritional Sciences, Texas Tech University, Lubbock, Texas, United States of America; All India Institute of Medical Sciences, INDIA

## Abstract

**Introduction:**

Eating habits have changed significantly over the past few decades, with consumers favoring unhealthy snack foods and beverages over traditional foods. This trend has shifted dietary patterns in developed and industrialized countries. However, this trend now also affects low- and middle-income countries, such as Nepal. Nepal is facing poverty, food insecurity, undernutrition like stunting and wasting, and also overweight and obesity during recent years among children and adolescents. The primary aim of this review is to assess the association between the consumption of unhealthy snack foods and beverages and the nutritional status of children and adolescents in urban, peri-urban, and rural Nepal.

**Materials and methods:**

Electronic databases like PubMed, Google Scholar, Web of Science, and Scopus were used to undertake a thorough desk-based searching of the literature. The literature was chosen using a number of inclusion and exclusion criteria. The selected publications were gathered, appraised for quality assessment, and results synthesized from studies.

**Results:**

Unhealthy snack food and beverages consumption is frequently reported to be high in urban areas and is significantly associated with higher odds of overweight or obesity. In sub-urban areas, the consumption is at a moderate level, exceeding that of rural areas, while generally lower than in urban settings and comparatively lower among rural youngsters, however, marketing initiatives and increased access are steadily raising the consumption. Although the majority of Nepalese children maintain a normal weight, underweight and stunting remain significant public health issues, and overweight and obesity are growing issues that require prompt action.

**Conclusions:**

Children and adolescents in Nepal face two nutritional challenges: a growing prevalence of overweight and obesity and ongoing undernutrition. Undernutrition and stunting are major problems in rural Nepal, and the widespread consumption of unhealthy snack food and beverages in urban areas is strongly associated with the rising prevalence of overweight and obesity. These issues reflect the ongoing differences in food diversity, accessibility, and overall dietary quality between rural and urban settings.

## 1. Introduction

Nutritional status is the state of health influenced by the intake and utilization of nutrients. According to the World Health Organization (WHO), malnutrition now encompasses a double burden consisting of both undernutrition (wasting, stunting, and underweight) and over-nutrition (overweight and obesity) [1,2]. Globally, this has reached critical proportions; in 2024, approximately 35 million children under five were overweight or obese, while 45 million were wasted and 149 million were stunted [3]. According to recent estimates, the number of obese people worldwide is quickly surpassing one billion before 2030 [4]. While traditionally viewed as a crisis of affluent Western nations like the United States and United Kingdom [5], this nutritional imbalance is rapidly shifting toward developing regions.

In Asia, the transition is particularly stark. By 2024, Asia accounted for nearly half of all overweight or obese children under the age of five globally [3]. This regional shift largely coincides with a dramatic 23.6% global increase in the per capita number of chain supermarkets and convenience stores over the last 15 years, a trend that disproportionately impacts low- and middle-income nations [6]. As these retail environments expand, traditional handcrafted diets are being replaced by mass-produced, calorie-dense products [7,8]. This transition is increasingly evident in Nepal, a developing low-middle income South Asian nation, currently facing the Double Burden of Malnutrition (DBM) [9]. National data highlights the complexity of this burden: while the Nepal Demographic Health Survey (NDHS) 2022 shows that 25% of children under five are stunted and 19% are underweight, there is a rising trend of excess weight among older cohorts [10]. Among adolescents aged 10–19, overweight prevalence has reached 5% in boys and 4% in girls [11]. This is a significant public health concern, as 70% of obese adolescents and 30% of overweight children are likely to remain obese into adulthood, carrying associated psychological and physical morbidities [12,13].

The prevalence of Unhealthy Snack Food and Beverage Consumption (USFBC) has increased significantly in Nepal, and is increasingly associated with the rising burden of childhood obesity and stunting [14]. A primary driver of this shift in Nepal is the rise of junk food, defined as discretionary items high in energy, fats, and sugars but low in essential micronutrients [15]. In Nepal, dietary patterns were significantly altered by events such as the 2015 earthquake, which accelerated the consumption of unhealthy snacks due to increased vending establishments and the inclusion of packaged unhealthy foods in relief provisions [16]. Recent studies in urban Nepal show that a majority of beverages and snacks consumed by children aged 12–23 months are nutritionally poor [17]. Among adolescents, processed food consumption is remarkably high, with 60.3% of students in the Kaski area reporting regular intake over nutritious alternatives [18,19].

This review focuses on the consumption of Ultra-Processed Foods (UPF) – defined by the NOVA classification as industrial formulations of food-derived substances typically containing five or more ingredients, such as hydrogenated oils, dyes, and flavor enhancers [20]. Despite the clear rise in both malnutrition and ultra-processed food availability in Nepal, no prior research has comprehensively synthesized the intertwined relationship between these two factors. Nepal serves as a critical representative model for other South Asian nations – such as India, Pakistan, and Bhutan, facing similar socio-demographic transitions. Therefore, the primary objective of this review is to evaluate the association between unhealthy snack food and beverage consumption, and the nutritional status of children and adolescents in Nepal.

## 2. Materials and methods

### 2.1. Study design

This systematic review was conducted in accordance with the Preferred Reporting Items for Systematic Reviews and Meta-Analyses (PRISMA) guidelines [21]. However, a formal review protocol was not prospectively registered in any public database (such as PROSPERO). The study utilized a comprehensive desk-based methodology to identify and synthesize the evidence. The protocol was designed to ensure transparency and reproducibility, with all stages of study selection, data extraction, and quality appraisal performed manually and in duplicate by the authors to minimize bias.

### 2.2. Study selection criteria

**Inclusion Criteria:** Studies were eligible for inclusion if they met the following criteria:

- Study Design: Peer-reviewed, primary cross-sectional studies published between January 1, 2019, and December 31, 2025.
- Population: Children and adolescents (aged 0–19 years) residing in Nepal.
- Exposure: Studies examining the consumption of commercially prepared, nutrient-poor foods and beverages. This included operational definitions across the literature encompassing unhealthy snack foods, junk foods, ultra-processed foods (UPFs), packaged snacks, sugar-sweetened beverages (SSBs), and fast foods.
- Outcome: Assessment of nutritional status, including undernutrition (stunting, wasting, underweight) or overnutrition (overweight, obesity).

**Exclusion Criteria:** Studies were excluded if they met any of the following criteria:

- Timeline: Published prior to January 1, 2019, to ensure findings reflect Nepal’s contemporary nutrition transition and current food environment.
- Geography: Regional, multinational, or global studies that did not provide a distinct, disaggregated, and extractable dataset specifically for the Nepalese population.
- Methodology: Longitudinal (cohort), case-control, and interventional study designs were excluded to maintain methodological homogeneity.
- Publication Type: Systematic reviews, meta-analyses, case reports, editorials, commentaries, and conference abstracts lacking full methodological detail or peer-reviewed full texts.

### 2.3. Search strategy

A systematic and comprehensive search of four electronic databases; PubMed, Scopus, Web of Science, and Google Scholar was conducted in accordance with PRISMA 2020 guidelines and checklist (Supplementary: S1 File). Regional and indigenous database – NepJOL was not used as a strategic choice to avoid duplicate records, noting that Nepalese health journals are already indexed in primary databases like PubMed/Scopus/Google Scholar, ensuring no loss of eligible articles. Search strategy for each database, including Boolean operators; AND, OR; controlled vocabulary – MeSH terms like; “Nutritional Status”, malnutrition, children; Title/Abstract Tag (tiab), Title and Abstract Keyword (TITLE-ABS-KEY), Title Search operator (TS); keywords and limits, were applied. The core search concepts were: unhealthy snack food (ultra-processed food, junk food, fast food, and packaged food) and sugary beverages consumption, nutritional status (normal weight, underweight, stunting, wasting, overweight, and obesity), and children and adolescents in Nepal. The details of search strategy for all database is available in Supplementary S2 File.

Limits used in search strategy: In PubMed, we applied limits for publication dates (2019–2025), humans, and journal articles. In Google Scholar, only publication year limits (2019–2025) were applied. Web of Science was filtered by publication year (2019–2025), and document type (article). In Scopus, limits for publication year (2019–2025), and document type (article). Study design, date of publication, and variables-specific eligibility were applied during screening. All searches were run independently by two authors. The articles were finalized to include in the study after a discussion and reaching consensus based on the eligibility criteria.

### 2.4. Compilation of articles

Search results were exported to and managed using Zotero reference management software [22]. Zotero was used to organize, compile, and deduplicate the articles. It assisted in screening the titles and abstracts of articles. Zotero was also used for the ease of referencing and citation.

### 2.5. Quality assessment

Study quality was assessed using the Joanna Briggs Institute (JBI) Critical Appraisal Checklist for Analytical Cross-Sectional Studies [23]. The checklist evaluated eight domains of questions and each study was scored as “Yes, No, Unclear, or Not applicable” for each domain (Supplementary S3). These eight domains are: were the criteria for inclusion in the sample clearly defined?, were the study subjects and the setting described in detail?, was the exposure measured in a valid and reliable way?, were objective, standard criteria used for measurement of the condition?, were confounding factors identified?, were strategies to deal with confounding factors stated?, were the outcomes measured in a valid and reliable way?, and, was appropriate statistical analysis used?

JBI does not recommend a scoring system to assess the quality because not all questions are equal and they are designed primarily for qualitative judgment. So, studies were ranked as low, moderate and high risk based on their adherence to critical methodological domains. Studies lacking clear strategies for confounding control or detailed participant descriptions were categorized as high risk. Moderate risk was assigned to studies that met most criteria but utilized convenience sampling or small sample sizes, potentially limiting generalizability. Low risk was reserved for studies demonstrating high methodological rigor, including the use of validated exposure tools and advanced statistical control for confounding.

Two authors independently appraised each included article; Discrepancies were resolved through discussion until consensus was reached. The reporting of quality assessment, available in the results below, adheres to PRISMA directives requiring transparency in risk-of-bias assessment.

### 2.6. Data extraction, analysis, and synthesis

Data extraction from the articles were performed separately by the two authors, followed by their comparison, noting the discrepancies, discussion and fixing the discrepancies. For each eligible study, we extracted study details (author & date), area of study (study setting), sample size, age of sample population, nutritional status sssessment tool, dietary assessment tool, nutritional status, and unhealthy snack food and beverages consumption practices/association.

Study settings were classified based on Nepal’s federal administrative boundaries under the Local Government Operation Act, 2017 and official definition with the contextual reality [24,25]. Metropolitan and Sub-metropolitan cities were categorized as urban; Municipalities were categorized as semi-urban; and Rural Municipalities were categorized as rural. Studies encompassing entire districts or a combination of different administrative tiers were categorized as Mixed settings to accurately reflect the socio-demographic diversity of the study population.

Data were presented in terms of percentage, range, and odds ratio to compare the quantative outcomes. A narative synthesis was performed to describe findings on unhealthy snack food and beverages consumption.

## 3. Results

### 3.1. Search results

**Fig 1:**
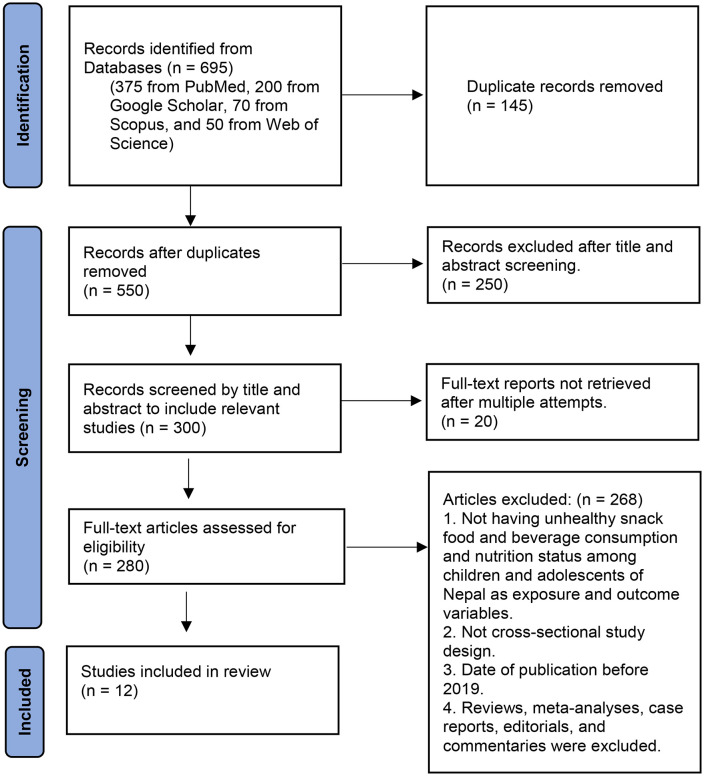
PRISMA flow diagram.

The initial database search yielded a total of 695 records. After the removal of duplicates and a multi-stage screening process for the eligibility (detailed in Fig 1), 12 primary research articles met all the eligibility criteria and were retained for the final synthesis. The included studies provide a comprehensive geographic representation of Nepal. Of the 12 studies, six were conducted in urban settings, three in peri-urban or sub-urban areas, and one in a rural region. Additionally, two studies included mixed participants from both urban and rural areas. This distribution ensures that the review captures the nutritional landscape across various socio-demographic environments within the country.

### 3.2. Quality assessment

Based on the JBI Analytical Cross-sectional Studies Checklist, risk of bias were categorized as low, moderate, and high. Details of the quality assessment is available as Supplementary S3 Table 1 below shows the overall quality assessment of studies.

**Table 1:** Reporting of quality assessment results.

Study (Author and Date)	Risk of Bias	Comments
Baral et al. (2025) Bhattarai & Bhusal (2019) Shrestha et al. (2021) Thapa et al. (2021) Pries et al. (2019)	Low	These studies utilized validated exposure tools, objective outcome measurements, large sample sizes, and advanced statistical control for confounding factors.
Bhatta et al. (2024) Karki et al. (2019) Pant (2021)	Moderate	These studies typically utilized restricted sampling (e.g., only private schools or specific grades) or had smaller sample sizes, which may limit generalizability or statistical power.
Khadka et al. (2020) Sharma et al. (2025) Tsang et al. (2019) Zahid et al. (2020)	High	These studies failed to provide sufficient detail on sample characteristics or strategies to address confounding, significantly weakening the confidence in their reported associations.

### 3.3. Study outcomes

#### 3.3.1. Nutritional Status of Children and Adolescents in different Settings (Urban vs Peri-urban vs Rural vs Mixed; mix of urban and rural).

Table 2 below presents the nutritional status of children and adolescents in different settings with their sample size and age group.

**Table 2:** Nutritional status of Nepalese children and adolescents.

Study	Area	Sample Size	Age group (years)	Weight (%)			
				Normal	Underweight	Overweight	Obese
Pant, 2021	Urban	100	3-5	67%	33%	–	–
Baral et al., 2025	Urban	460	16-19	71.90%	8.50%	19.60%	–
Bhatta et al., 2024	Urban	240	10-13	56.00%	44.00%	–	–
Karki et al., 2019	Urban	575	6-13	63.60%	10.7%	18.60%	7.10%
Bhattarai & Bhusal, 2019	Urban	510	14-17	74.30%	21.80%	3.10%	0.80%
Pries et al. 2019	Urban	745	1-2	Stunting – 18.8% and Wasting – 5%			
Shrestha et al., 2021	Rural	708	8-16	27% (Stunting)			
Zahid et al., 2020	Rural & Urban	273	0.5 - 12	21% (Stunting), 14% (Underweight), 6% (Wasting)			
Tsang et al., 2019	Rural & Urban	836	0.5 - 6	33% (Stunting), 15% (Underweight), 2% (Wasting)			
Sharma et al., 2025	Peri-urban	678	15-19	47%	41%	8%	4%
Thapa et al., 2021	Peri-urban	422	5-18	–	–	8.20%	1.80%
Khadka et al., 2020	Peri-urban	100	< 5	44%	43%	11%	2%

Malnutrition (underweight, stunting, wasting) is significant in rural areas of Nepal and among the age groups of under 5 years whereas overweight & obesity is rising among age groups of 13–19 years in urban areas. The prevalence of normal weight exhibited the widest reported range (44%–74%), indicating that the majority of Nepalese children fall within this category. In contrast, the prevalence of underweight demonstrated substantial variability, ranging from approximately 8.5% to 44%, which reflects marked heterogeneity across studies and geographic settings. Stunting also remained notably high, between 21% and 33%. Overweight and obesity were reported at comparatively lower levels, roughly 3% to 20% and less than 1% to 7%, respectively, suggesting these conditions are emerging but still less common. Wasting represented the least prevalent form of malnutrition, with reported rates between 2% and 6%.

#### 3.3.2. Unhealthy Snack Food and Beverages Consumption among Children and Adolescents in different Settings.

Table 3 below shows the unhealthy dietary practices among children and adolescents of Nepal with the dietary assessment tools used in each study.

**Table 3:** Unhealthy snack food and beverages consumption among Nepalese children and adolescents.

Study	Study Area	Dietary Assessment Tool	USFBC Practices/ Association
	(Settings)		
Pant, 2021	Urban	Qualitative interview	Prevalence of USFBC was 48%.
Baral et al., 2025	Urban	Structured questionnaire	Daily consumption of USFBC was significantly associated with higher odds of being overweight compared to consumption ≤ twice a week (aOR = 3.1, 95% CI: 1.5–6.6).
Bhatta et al., 2024	Urban	Self-administered questionnaires	A significantly higher proportion of underweight status was observed among those consuming junk food (p = 0.013).
Karki et al., 2019	Urban	SPANS 2010 questionnaire translated and adapted to the Nepalese context	Consuming USFBC more than twice a week was associated with higher odds of overweight or obesity (aOR = 2.9, 95% CI: 1.6–5.1).
Bhattarai & Bhusal, 2019	Urban	Pretested semi structure self-administered questionnaire	Daily consumption of USFBC was reported by 57.6% of respondents.
Pries et al. 2019	Urban	Quantitative 24-h recalls	Higher consumption of processed snacks was significantly correlated with a higher prevalence of childhood stunting.
Shrestha et al., 2021	Rural	Food Frequency Questionnaire (FFQ) and Principal Component Analysis (PCA)	Higher USFBC consumption scores were associated with children whose caregivers were engaged in outdoor occupations.
Zahid et al., 2020	Rural & Urban	Interview responses of parents/caregivers recorded on iPads via the 2015 Qualtrics platform (Provo, UT, USA)	Dental caries was identified as a significant mediating factor linking junk food consumption and poor nutritional outcomes.
Tsang et al., 2019	Rural & Urban	Interviewed mothers or caregivers using a survey instrument modified from the World Health Organization (WHO)	Urban children over age 3 consumed ultra-processed items at double the rate of rural children; both patterns were strongly associated with stunting and underweight.
Sharma et al., 2025	Peri-urban	Global Dietary Recommendations (GDR) Score derived from the Diet Quality Questionnaire (DQQ)	The absence of home starchy staple foods the previous day was associated with a lower BMI (−5.58 units) compared to those with availability.
Thapa et al., 2021	Peri-urban	Pretested set of semi-structured questionnaire	Over one-third of children with overweight/obesity were identified as frequent consumers of unhealthy snack foods and sugary drinks.
Khadka et al., 2020	Peri-urban	Pre-tested questionnaire	A significant, positive linear correlation was observed between feeding practices and child anthropometry.

The findings highlights a critical dietary transition in Nepal where Unhealthy Snack Food and Beverages Consumption (USFBC) have become a primary driver of the Double Burden of Malnutrition. In urban settings, where daily consumption exceeds 50%, children are twice as likely to consume ultra-processed items compared to rural peers, leading to a significant health paradox: frequent consumption (more than twice weekly) triples the risk of overweight and obesity (aOR ≈ 3.0), yet simultaneously correlates with stunting and underweight status due to nutrient displacement. This nutritional crisis is further compounded by a “missing link” of dental caries and is increasingly penetrating rural and peri-urban areas, where caregiver occupation and food availability significantly dictate poor anthropometric outcomes across all age groups.

## 4. Discussion

Studies from urban area indicates an early onset of unhealthy dietary behaviors even in preschool-aged children, higher exposure and access to unhealthy packaged food, and rising rates of childhood overweight/obesity. The predominance of ultra-processed food consumption across all urban settings underscores the shifting dietary habits influenced by increased availability, affordability, and preference for unhealthy snack foods among younger populations [26–31]. Only one study, among twelve studies in our systematic review, was carried out entirely in rural Nepal. This study indicated strong methodological rigor during the quality assessment and lending high confidence to the validity of its findings. The study demonstrated that socioeconomic factors, particularly caregiver employment and associated income, may influence dietary behaviors, potentially increasing household access to packaged or convenience foods even within rural settings [32]. This underscores the complex interplay between socioeconomic conditions, food availability, and dietary choices among school-aged children. Mixed-setting studies, having participants from both urban and rural area, reinforce the dual burden of malnutrition in Nepal: undernutrition remains a major concern, particularly in rural communities, while urban children increasingly encounter obesogenic environments that promote unhealthy dietary practices [33,34]. Studies from peri-urban area indicate that limited access to healthy food options and high availability of unhealthy packaged foods contribute to increased consumption of unhealthy items and higher risks of over-nutrition, whereas access to home-prepared foods is associated with healthier dietary patterns and lower BMI [35–37].

Earlier systematic reviews, though largely global in scope, consistently reported positive associations between consumption of sugar-sweetened beverages (SSBs), ultra-processed foods (UPFs), and fast food with increased adiposity among children and adolescents. An earlier systematic review evaluating the association between SSB consumption and weight gain, overweight, and obesity in a population of 6-month-old to 19-year-old children and adolescents found that higher SSB intake increases the risk of overweight and obesity [38]. Another similar systematic review and meta-analysis study identified significant pooled effects of SSB consumption on BMI in children, reinforcing the strength of these associations [39]. Reviews on ultra processed foods and fast-food intake further support these conclusions, demonstrating that unhealthy, energy-dense dietary patterns are strongly associated with higher BMI and adiposity among youth populations globally [40,41]. Compared with these earlier reviews, our findings are consistent regarding the positive association between USFBC intake and higher overweight/obesity, particularly in urban regions. What distinguishes our review is its focus on geographical context – urban, peri-urban, and rural areas, which provides a more nuanced understanding of how environmental exposure and food environments shape dietary behavior. While global reviews lump populations together, our analysis reveals heterogeneity within the same country context, particularly in peri-urban regions where dietary transition is underway. This mid-transition zone, rarely highlighted in global reviews, shows rising junk-food exposure but still retains risks of undernutrition, a dual burden not emphasized in previous literature.

Our review further illustrates how structural determinants, such as commercialization of foods, school canteen policies, and aggressive marketing, shape dietary preferences and are linked to a higher prevalence of obesity in urban youth. These insights align with WHO-commissioned meta-analysis and evidence syntheses reporting that unhealthy food environments are associated with elevated BMI in children [42]. In contrast, our study showing the minimal link between USFBC intake and nutritional outcomes in rural settings reflects limited access to processed foods and retention of traditional diets. This differs from global studies that generally do not differentiate rural food environments. A recent article from Nutrition Reviews on UPFs consumption strongly supports that UPF intake in youth is associated with poor diet quality, overweight/obesity, and related health risks [43]. Additionally, recent meta-analyses focused on UPF and obesity risk, and fast-food restaurants with overweight and obesity in school-aged children and adolescents provided a quantified pooled estimate – reinforcing that high UPF intake correlates with greater obesity risk [44,45].

This systematic review provides a foundational framework to initiate the pattern of integrated analysis of USFBC and nutritional status among children and adolescents in Nepal. This review only selected cross-sectional studies as they are the primary design used to measure population level prevalence and simultaneous exposure-outcome relationships, which aligns with the objectives of this review. Moreover, mixing study designs could lead to the inconsistency of result synthesis and interpretation which this study avoided completely. A key strength of our review is its contextual comparative approach, which offers more actionable insights for policy-makers and program planners, especially in settings undergoing rapid nutrition transition. By categorizing studies by urbanicity, we highlight heterogeneity that has been poorly recognized in earlier global syntheses. This systematic review serves as a foundational investigation, aiming to establish a preliminary evidence base and articulate a clear research gap. The findings are anticipated to stimulate scholarly inquiry and catalyze future research focused on underrepresented nexus of dietary behavior and health outcomes.

The precision and confinement of the review’s inclusion criteria resulted in a highly restricted sample size of relevant literature. This consequently led to the scarcity of data which underscores the fact that, while the overarching field possesses significant scope and critical public health relevance, the specific intersection of these two key variables (USFBC and Nutritional Status) remains insufficiently explored by the academic community. Also restricting this review to cross-sectional studies may limit casual inference. Another limitation of this review is the restriction to studies published from 2019–2025. This window was selected deliberately to capture the most contemporary evidence. However, it may miss longer-term baseline trends and earlier studies that would improve historical context and trend estimation. The lack of standardized age classifications in the primary literature introduced challenges in demographic categorization as children and adolescents.

Our synthesis revealed a stark geographic imbalance in the available literature, with the vast majority of studies focused on urban (n = 6) and peri-urban (n = 3) settings, compared to only one purely rural study. In settings like Tsang et al. [34], urban children were shown to consume ultra-processed foods at twice the rate of their rural counterparts. This imbalance reflects the rapid commercialization and changing food environments in Nepal’s municipal areas. However, it also means that the overall heavily documented association between USFBC and poor nutritional outcomes (such as overweight trends in Baral et al. [27] and stunting patterns in Pries et al. [31]) may underrepresent the unique dietary challenges faced by rural, isolated populations where food insecurity and traditional undernutrition overlap.

## 5. Conclusion and recommendations

The synthesized evidence highlights a dual nutritional challenge among Nepalese children and adolescents, characterized by persistent undernutrition and a rising prevalence of being overweight and obese. The high prevalence of unhealthy snack food and beverage consumption in urban settings is strongly associated with overweight and obesity outcomes, highlighting how dietary patterns are changing due to the availability, cost, and younger populations’ preference for fast food. Although ultra-processed food consumption is comparatively lower in rural Nepal, undernutrition and stunting remain significant challenges, reflecting persistent disparities in food diversity, accessibility, and overall dietary quality between rural and urban settings. These geographical disparities underscore Nepal’s ongoing nutrition transition, a process where modernization and urbanization are closely linked to the emergence of obesogenic environments within cities, while rural communities concurrently continue to contend with persistent nutrient deficiencies and food insecurity. The evidence underscores the need for multifaceted interventions: in urban areas, strategies should prioritize reducing exposure to unhealthy packaged foods and sugar-sweetened beverages and promoting balanced diets, while rural and peri-urban programs should focus on improving food diversity, monitoring growth, and mitigating undernutrition, alongside policies addressing food marketing targeted at children.

The consistency between our findings and earlier global reviews reinforces the urgent need for environmental and policy interventions, especially in urban and peri-urban settings. School-based regulation of unhealthy packaged food, high taxing sugar-sweetened beverages, school feeding programs in rural districts and home-made food as tiffin in urban districts instead of canteen in schools, nutritional education to caregivers/mother to reduce USFBC consumption, and promotion of healthier food environments could substantially mitigate rising overweight and obesity, and stunting trends among Nepalese children and adolescents. Future research should adopt: longitudinal designs to establish causal relationships, rural and peri-urban representation to understand emerging transitions and multilevel frameworks that capture environmental, behavioral, and socioeconomic drivers.

## Supporting information

S1 File PRISMA 2020 Checklist. (PDF)

S2 File Search strategy for each database, S3; Quality Assessment with JBI Appraisal Checklist. (PDF)

S3 File Supplementary File. (XLSX)
