# Supplementary material for: Association between unhealthy snack food and beverage consumption and the nutritional status of Nepalese children and adolescents: A systematic review
Source: PLoS One. 2026 Jun 25;21(6):e0352341. doi: 10.1371/journal.pone.0352341 (PMC13298750; doi:10.1371/journal.pone.0352341)
Supplement: S2 File — (PDF) [file pone.0352341.s002.pdf]

Supplementary: Database Search Strategies

PubMed
(“junk food”[tiab] OR “fast food”[tiab] OR “ultra-processed food”[tiab]
OR “packaged food”[tiab] OR “snack food”[tiab] OR “sugary beverages”[tiab])
AND (“Nutritional Status”[MeSH] OR “malnutrition”[MeSH] OR underweight[tiab]
OR overweight[tiab] OR obesity[tiab] OR stunting[tiab] OR wasting[tiab])
AND (children[MeSH] OR child*[tiab] OR adolescent*[tiab] OR “school-aged”[tiab])
AND (Nepal[tiab])

Limits: publication date (2019–2025), humans, and journal articles.

Scopus
TITLE-ABS-KEY (“junk food” OR “ultra processed food” OR “snack food”
OR “packaged food” OR “fast food” OR “sugary beverages”) AND TITLE-ABS-KEY
(obesity OR overweight OR malnutrition OR underweight OR stunting OR wasting)
AND TITLE-ABS-KEY (child* OR adolescent* OR “school-aged”) AND TITLE-ABS-KEY (Nepal) AND PUBYEAR > 2018

Limits: publication year (2019–2025), document type (article)

Web of Science
TS=(“junk food” OR “ultra processed food” OR “snack food” OR “packaged food” OR
“fast food” OR “sugary beverages”) AND TS=(obesity OR overweight OR malnutrition
OR underweight OR stunting OR wasting) AND TS=(child* OR adolescent* OR
“school-aged”) AND TS=(Nepal)

Limits: publication year (2019–2025), document type (article)

Google Scholar
"junk food" AND Nepal AND "nutritional status"

"fast food" AND Nepal AND obesity

"ultra-processed food" AND Nepal AND stunting

"sugary beverages" AND Nepal AND children AND malnutrition
(First 200 results screened per PRISMA-s compliant guidance).

Limits: publication year (2019–2025)
