# Supplementary material for: Association between unhealthy snack food and beverage consumption and the nutritional status of Nepalese children and adolescents: A systematic review
Source: PLoS One. 2026 Jun 25;21(6):e0352341. doi: 10.1371/journal.pone.0352341 (PMC13298750; doi:10.1371/journal.pone.0352341)
Supplement: Attachment — Submitted filename: Response-letter-Updated.pdf [file pone.0352341.s004.pdf]

Dear Editorial office,
PLOS One
Resubmission of PONE-D-25-61915

We appreciate your time and making the effort to review the paper and point out some issues during earlier submission. Please see the responses below.

Comments	Response	Page and Point (Changes visible)
We do not feel the tool used for the quality assessment or risk of bias is suitable for the studies included in your review.	Thank you for the comments.<br><br>We have now used the most suitable quality appraisal checklist (JBI) for reporting risk of bias for all included studies. A supplementary file with checklist adds more clarity and methodological quality in this systematic review study.<br><br>Significant changes can be seen at:<br>♦ Search strategy<br>♦ Quality Assessment<br>♦ Results of quality assessment	♦ Page – 6 and Point – 2.3<br>♦ Page – 7 and Point - 2.5<br>♦ Page – 8 and Point – 3.2
Upload copies of the completed PRISMA checklist as supporting information and a flowchart as Figure 1.	PRISMA flow diagram now uploaded as Figure 1 as asked by the Technical Check Team. Earlier, I had inserted the diagram in the Manuscript.<br>Completed PRISMA checklist is available as supporting information and uploaded during the submission.	

Thank you for going through this letter. We hope this letter along with the revised manuscript will address your concern.

Sincerely,

Author of the manuscript
