# Supplementary material for: Association between unhealthy snack food and beverage consumption and the nutritional status of Nepalese children and adolescents: A systematic review
Source: PLoS One. 2026 Jun 25;21(6):e0352341. doi: 10.1371/journal.pone.0352341 (PMC13298750; doi:10.1371/journal.pone.0352341)
Supplement: Attachment — Submitted filename: Response to Reviewers.pdf [file pone.0352341.s005.pdf]

Dear Editor,
PLOS ONE
[PONE-D-25-64936]

Thank you for your precious time and effort to help us improvise the manuscript with your valuable comments and concern. We truly acknowledge your contribution. Kindly see the responses below.

Comments and Suggestions	Response by the author	Changes Visible
Limitations in the search strategy and study selection (including exclusion of many full texts)	Thank you for this methodological concern. To address this, we re-ran the search strategy and conducted a thorough audit of the records initially categorized as inaccessible. We discovered that the vast majority of these 150 records were ineligible for inclusion: many were review articles, published before our 2019 cutoff, or focused on variables other than the specific link between Unhealthy Snack Food and Beverages Consumption (USFBC) and nutritional status. Only one additional eligible study was identified and has now been included in the synthesis. We have updated our PRISMA flow diagram and the Results section to reflect these changes.	Line: 155 – 165<br><br>PRISMA Flow Diagram – Figure 1
Insufficient methodological transparency (registration, eligibility criteria, and risk-of-bias assessment)	While this specific review was not prospectively registered in a public database like PROSPERO, we strictly adhered to a pre-defined internal protocol and the PRISMA 2020 guidelines to ensure methodological rigor. To ensure transparency, we have provided the completed PRISMA checklist as Supplementary Material S1, which details our adherence to the established standards of systematic reporting.<br><br>Regarding eligibility criteria and risk-of-bias assessment, we have thoroughly addressed your concern at Section 2.2 and 2.5 by clarifying the exclusion criteria further and elaborating the JBI criteria.	Supplementary File – S1 – PRISMA Checklist 2020<br><br>Line: 91 – 97 & 123 - 142

Over interpretation of findings derived from cross-sectional studies<br><br>Strengthened data synthesis (including stratification where feasible)<br><br>Contextually grounded discussion and policy implications	Both results and discussion revised.<br><br>We concise our results with stratification.<br><br>Discussion is now more precise.<br>Policy implications are now more grounded towards Nepal context.	 <br>Line: 172 – 206<br><br>Line: 207 - 259<br>Line: 288 - 292
The manuscript would benefit from improved structure and clarity	Yes, we totally agree. An improved result section, distinct discussion, elaborated and clarified quality assessment have now addressed your concern.	Line: 123 – 142 & 172 – 259
Clearer definitions of exposure (junk food vs. ultra-processed foods)	We have included NOVA definition for ultra-processed food and distinguish it from junk food with citation added. We have also re-titled our manuscript to avoid any confusion among readers. Search strategy for core concepts is also further clarified.	Title Revised<br><br>Line: 69 – 71 & 107 - 110

Note from the authors: In this revised manuscript, we have included Dr. Surya Raj Niraula as a co-author to reflect his substantial intellectual contribution to the revision process. As a Biostatistician, Dr. Niraula performed a comprehensive ratification and audit of the extracted data, ensuring the statistical consistency of nutritional metrics across the heterogeneous study designs. Dr. Niraula contributed in re-screening of the inaccessible full-text articles and re-audit them. His expertise was instrumental in the structural re-organization of the results, specifically in refining the data presentation to improve clarity and accuracy. These contributions have significantly strengthened the methodological framework and the overall scientific integrity of the review.

Thank you for going through this response letter. We hope this letter along with the revised manuscript will address all your valuable comments.

Sincerely,

Author of the manuscript

Dear Reviewer1,
PLOS ONE
[PONE-D-25-64936]

Thank you for your precious time and effort to help us improvise the manuscript with your valuable comments and concern. We truly acknowledge your contribution. Kindly see the responses below.

Comments and Suggestions	Response by the author	Changes Visible
One limitation is the time frame restriction (2019–2025), which is relatively short for a systematic review and could introduce selection bias by excluding relevant evidence published in earlier periods.	We acknowledge the Reviewer's concern regarding the search timeline (2019–2025). This window was selected to capture the most contemporary evidence following the rapid expansion of the processed food industry in Nepal. Given the speed of the nutrition transition in South Asia, we believe that including studies older than 2019 would dilute the relevance of the findings for current policymakers. However, to address this concern, we have added a section in the Discussion justifying this timeframe and acknowledging it as a purposeful focus on the 'modern' dietary landscape.	Line: 266 – 270
150 articles were excluded due to lack of access to the full text. This considerable number represents a significant methodological weakness, as obtaining the full texts of potentially eligible studies is an expected part of the process in this type of research.	Thank you for this methodological concern. To address this, we re-ran the search strategy and conducted a thorough audit of the records initially categorized as inaccessible. We discovered that the vast majority of these 150 records were ineligible for inclusion: many were review articles, published before our 2019 cutoff, or focused on variables other than the specific link between Unhealthy Snack Food and Beverages Consumption (USFBC) and nutritional status. Only one additional eligible study was identified and has now been included in the synthesis. We have updated our PRISMA flow diagram and the Results section to reflect these changes.	Line: 155 – 165<br><br>PRISMA Flow Diagram – Figure 1

It is also observed that the studies included in the review come exclusively from a single country, which considerably limits the generalizability and international scope of the findings.	We acknowledge the reviewer's point regarding the geographic scope of the review. The primary objective of this study was to provide a rigorous and comprehensive synthesis of the evidence specifically within the context of Nepal—a nation currently undergoing a rapid and unique nutrition transition.<br><br>While we agree that the findings may not be directly generalizable to disparate global contexts, Nepal serves as a critical case study for low- and middle-income countries (LMICs) experiencing similar shifts in food environments.	
The abstract does not specify the search period or the inclusion criteria used.	To prepare a concise abstract we have not detailed inclusion criteria and search period in the abstract. We tried to include the summarized gist of methodology in the abstract.	
The introduction needs improvement in its logical sequence; it is disorganized, repeats ideas in different paragraphs, and does not delve deeply into aspects that could be useful in the discussion, thus hindering comprehension.	We have re-organized the introduction section in a funnel manner (global to regional to local). Thank you for this comment.	Line: 36 - 68
The methodology section does not detail the scoring system used to categorize studies as low, moderate, or high risk; it only states that the JBI was used.	Risk of bias was assessed qualitatively based on the JBI Critical Appraisal Checklist. JBI does not recommend the scoring system so, rather than using a numerical score, studies were categorized based on their adherence to critical methodological domains. Studies lacking clear strategies for confounding control or detailed participant descriptions were categorized as High Risk. Low Risk was reserved for studies employing validated measurement tools and robust statistical adjustments.	Line: 124 - 139

The results show a tendency to suggest causal relationships, even though the included studies are cross-sectional in design, which limits the conclusions.	We have now stratified our results with more clarity.<br><br>Limitations caused by cross-sectional studies and the alignment with objectives is clearly stated in the discussion section' limitation and strength.	Line: 172 – 206<br><br>Already acknowledged at 265 – 266 & 274 - 278
While the authors acknowledge some limitations - such as the exclusive inclusion of cross-sectional studies, the limited time frame, and the small number of studies - these should be discussed in greater depth considering their impact on the interpretation of the findings.	Thank you. We have addressed all limitation along with interpretation of results based on the quality assessment.	

Thank you for going through this response letter. We hope this letter along with the revised manuscript will address all your valuable comments.

Sincerely,

Author of the manuscript

Dear Reviewer2,
PLOS ONE
[PONE-D-25-64936]

Thank you for your precious time and effort to help us improvise the manuscript with your valuable comments and concern. We truly acknowledge your contribution. Kindly see the responses below.

Comments and Suggestions	Response by the author	Changes Visible
The gender distribution of participants in each included study should be reported, as dietary habits may differ between males and females.	We thank the reviewer for this important suggestion. We agree that gender is a critical determinant of dietary behavior. However, during our data extraction, we found that the majority of the included primary studies did not report outcomes or dietary habits (USFBC) separately for male and female participants. Most studies reported results for the pediatric and adolescent population as a single aggregate result.	
The specific types of junk food examined in each individual study must be clearly described. The current definition is overly broad, and detailed itemized food categories should be provided in the Results section.	We have revised our title of the manuscript to avoid confusion among the readers. In addition, we have included NOVA definition for ultra-processed food and distinguish it from junk food with citation added. Search strategy for core concepts is also further clarified.	Title Revised<br><br>Line: 69 – 71 & 107 - 110
Only 1 study was conducted exclusively in rural areas and 3 in peri-urban areas, leading to an imbalanced sample distribution across urban, peri-urban, and rural settings. Consequently, the conclusions regarding rural populations	We believe the stratified result section now address your concern.	Line: 172 - 206

lack credibility. It is strongly recommended that the authors explicitly acknowledge this sampling bias in the Results section and note the limited generalizability of rural findings. Alternatively, high-quality rural studies should be added.		
Figure 2, which presents the range of nutritional status percentages, is of low quality because it lacks error bars or confidence intervals. The figure should be revised to a bar chart stratified by urban, peri-urban, and rural areas.	Figure 2 has been removed during re-structuring of our result section.	Result Section
When synthesizing data, the authors should analyze the association between the frequency of junk food consumption and the risk of malnutrition (e.g., dose–response relationships).	We appreciate the reviewer's suggestion to explore dose-response relationships. We carefully re-evaluated the included studies to determine if such an analysis was feasible. However, we found that the primary literature lacks the granular, standardized data required for this type of synthesis. Specifically, the included studies utilized heterogeneous metrics for "frequency" (e.g., some used 24-hour recalls, others used food frequency questionnaires with different scales), and most reported consumption does not specify "dose" levels linked to outcome increments.<br><br>Consequently, a formal dose-response synthesis is not possible with the current evidence base.	
The topic of junk food intake and nutritional status in children is relatively conventional; however, the focus on the Nepalese population provides some novelty. The authors must	Title have been revised<br><br>Changes in the introduction (focus on Nepal earthquake, urbanization, and recent surge in development) to strongly establish the novelty of the study.	Title Revised<br><br>Line: 62 - 77

strengthen the discussion of local contextual factors in Nepal - including the earthquake, urbanization, food policies, and economic development - to more thoroughly explain urban–rural disparities. Without this, the novelty of the study cannot be sufficiently highlighted.		
In the Discussion section, the authors should address the biological and social mechanisms linking junk food to overweight/obesity and malnutrition in rural areas, such as high sugar/fat content, monotonous diets, and caregivers' nutritional knowledge.	Our revised discussion section has addressed your concern to some extent.	Line: 208 - 226
The current policy recommendations are too general. Specific, actionable, and context-appropriate measures should be proposed to improve practical utility.	Yes, we have now more grounded policy recommendations for Nepal-context like:<br><br>School-based regulation of unhealthy packaged food, high taxing sugar-sweetened beverages, school feeding programs in rural districts and home-made food as tiffin in urban districts instead of canteen in schools, nutritional education to caregivers/mother to reduce USFBC consumption, and promotion of healthier food environments.	Line: 288 - 293

Thank you for going through this response letter. We hope this letter along with the revised manuscript will address all your valuable comments.

Sincerely,

Author of the manuscript

Dear Reviewer3,
PLOS ONE
[PONE-D-25-64936]

Thank you for your precious time and effort to help us improvise the manuscript with your valuable comments and concern. We truly acknowledge your contribution. Kindly see the responses below.

Comments and Suggestions	Response by the author	Changes Visible
Was this systematic review registered?	While this specific review was not prospectively registered in a public database like PROSPERO, we strictly adhered to a pre-defined internal protocol and the PRISMA 2020 guidelines to ensure methodological rigor. To ensure transparency, we have provided the completed PRISMA checklist as Supplementary Material S1, which details our adherence to the established standards of systematic reporting.	Available file as - Supplementary File – S1 – PRISMA Checklist 2020
What is the current nutritional status of children and adolescents in Nepal? What are the common influencing factors? If there is a clear polarization, is it appropriate to focus solely on junk food?	In this review study we have addressed the Multifactorial Nature of Malnutrition (both under and over – nutrition).<br><br>While this review evaluates the link between Unhealthy Snack Food and Beverages Consumption (USFBC) and the nutritional status of children/adolescents, these findings must be interpreted within a multifactorial framework. Pediatric nutritional status in Nepal is shaped by a wide array of determinants beyond dietary intake, including socioeconomic status, access to clean water and sanitation (WASH), maternal nutritional literacy, and physical activity levels. USFBC likely acts as both a direct contributor to calorie excess and a marker for broader dietary shifts associated with urbanization and the global nutrition transition. Therefore, addressing the rise in USFBC should be viewed as one	

	critical component of a comprehensive public health strategy, rather than a singular solution to the complex challenge of malnutrition.	
Why were children and adolescents not analyzed separately? How did the authors account for puberty, which is a very important variable?	Thank you for this critical and significant comment.<br><br>The lack of standardized age classifications in the primary literature introduced challenges in demographic categorization as children and adolescents.	Line: 270 - 272
Is access to junk food the same for all children and adolescents? Furthermore, how do the prices of junk food compare to those of healthy foods or home-cooked meals? My concern here is whether economic factors might be driving children toward junk food consumption.	Our systematic review could not focus on every aspects of USFBC like prices of food and economic factors like pocket-money, income of family etc. However, we have addressed this as our recommendation to future research in Nepal to focus capturing environmental, behavioral, and socioeconomic drivers.	Line: 293 - 296

Thank you for going through this response letter. We hope this letter along with the revised manuscript will address all your valuable comments.

Sincerely,

Author of the manuscript

Dear Reviewer4,
PLOS ONE
[PONE-D-25-64936]

Thank you for your precious time and effort to help us improvise the manuscript with your valuable comments and concern. We truly acknowledge your contribution. Kindly see the responses highlighted below.

Abstract: The abstract states that the review aims to assess the relationship between children's nutritional status in Nepal and junk food consumption. However, the Results portion of the abstract focuses primarily on geographic distribution (urban, peri-urban, rural) rather than summarizing findings related to nutritional status outcomes. Consider revising the abstract to better reflect the stated aim by highlighting key nutritional status findings.

Response – Abstract have been revised.

Line: 7 – 9

Introduction: The overall structure of the introduction would benefit from greater consistency (e.g., presenting global context first, followed by regional and then Nepal specific data for each major theme).

- While the description of BMI and its association with overweight/obesity is factually correct, BMI alone is an imperfect proxy for nutritional status. A brief acknowledgment of its limitations (e.g., inability to distinguish body composition) would provide a more balanced framing.
- Beginning on line 57: Several prevalence statistics lack clear geographic context. For example: "In 2024, an estimated 35 million children under the age of 5 years were overweight and obese" — please specify whether this refers to global, regional, or country level estimates.
- Similar clarification is needed for statistics referring to Asia and for the figure stating that "more than 390 million children and adolescents aged 5–19 were overweight."
- Line 58: "The same inclining trend..." is introduced without a prior description of a trend; this should be revised for logical continuity.
- Line 60: "In 2024, Asia accounted for about half of all children under five 61 who were overweight or obese" could be read as referring either to the global distribution of overweight/obese children, or to the prevalence within Asia itself. Clarifying the intended interpretation would improve clarity.
- Line 75: The statement noting that 70% of obese adolescents and 30% of overweight children become obese adults is compelling and would be more effective if introduced earlier to motivate the public health relevance before presenting prevalence statistics.
- The discussion of undernutrition focuses largely on short term consequences (e.g., mortality under age five). Consider briefly addressing long term consequences (health,

economic, etc.) of childhood undernutrition to further strengthen the argument of why this is important.

- Line 88: The shift from under and over nutrition to eating patterns and junk food consumption is abrupt. The introduction would benefit from a clearer conceptual bridge explicitly linking dietary patterns (including junk/ultra processed foods) to both under and over nutrition.
- Line 104: The paragraph beginning here is considerably more detailed than necessary. Consider front loading the key takeaway (i.e., dietary shifts toward packaged convenience foods in Nepal) and then briefly summarizing supporting evidence, rather than presenting detailed study descriptions.

Response: We have thoroughly re-organized the introduction section in a funnel manner (global to regional to local). Thank you for this comment.

Line: 36 - 68

Methods:

- Section 2.1: This section repeats methodological details that are described more fully in subsequent subsections (e.g., search strategy, eligibility criteria, screening, data extraction). It could be streamlined to briefly state the review design and PRISMA adherence, with operational details retained in Sections 2.2–2.6.

  Response: Yes, we have now removed the redundancy in this section and revised it accordingly.
  Line: 81 – 86

- Section 2.2: The exclusion criteria largely mirror the inverse of the inclusion criteria, which limits their usefulness for understanding screening decisions. Clarifying explicit exclusion rules (e.g., mixed age samples without separable child data, multi country studies without Nepal specific results, non–peer reviewed publications) would strengthen methodological transparency.

  Response: Exclusion criteria are now more clarified to understand the screening decisions.
  Line: 91 – 97

- Section 2.4: Please provide a citation for the reference management software (Zotero).

  Response: Citation provided [22] with reference added as well.
  Line: 120

- Section 2.6: The description of extracted variables could be streamlined to emphasize analytically meaningful elements. Leading with bibliographic information adds little methodological clarity, as these details are implicit in any systematic review. Additionally, given that only cross sectional studies were eligible, it is unclear what analytic purpose

extracting "study design" served. Clarification or revision to focus on variables informing synthesis (e.g., exposure and outcome measurement, confounding, statistical approaches) is suggested.

Response: Thank you for the wonderful suggestion. Your comments have been addressed to some extent.
Line: 146 - 151

Results:

- Section 3.1: The step-by-step narrative of record counts duplicates information shown in the PRISMA flow diagram. For clarity and concision, the authors may wish to let Figure 1 present the detailed screening numbers and retain only a brief summary of the final inclusion in the text.

  Response: The repeated information has been removed and the search results have been revised accordingly.
  Line: 156 – 163

- Table 1: Table 1 is helpful, but the rationale for the overall risk-of-bias ratings would benefit from greater transparency and consistency. The brief narrative comments vary in focus and do not clearly indicate which JBI checklist domains most strongly influenced each appraisal. I suggest standardizing comments to explicitly reference key checklist items (e.g., exposure measurement, confounding control, sampling, outcome assessment) or briefly noting the primary domains driving the Low/Moderate/High rating. This would improve interpretability and comparability across included studies.

  Response: The revised Quality assessment section – 2.5 and re-structured quality assessment table have addressed your concern.
  Line: 124 – 139

- Lines 240-245 and Table 2: The description of the narrative synthesis and Table 2 is awkwardly worded and somewhat unclear. Phrases such as "summarized and analysed narratively by presenting them in the below table" are redundant and imprecise, and the listed table domains mix bibliographic details, methods, and findings without a clear organizing rationale. In addition, the justification for not conducting statistical analyses is not strictly necessary given the absence of meta-analysis. Streamlining this section to clearly describe the contents and purpose of Table 2, and briefly justify the use of narrative synthesis, would improve readability and methodological clarity.

  Response: Result section 3.3 is now stratified to make our findings clearer and precise. Narrative synthesis have been clearly stated in the section 2.6.
  Line: 149 – 151 & 173 – 197

- Figure 2: The table title refers to “Children,” while the caption refers to “Children and Adolescents.” Please clarify which population is represented and specify the age range. If feasible based on available data, stratifying by age group may strengthen this figure.

  Response: Figure 2 is now removed during the re-structuring of our result section.

- Lines 277–287: This paragraph contains several interpretive claims without explicit citations. Even if these statements reflect synthesis by the authors, referencing the specific included studies that support each point would strengthen credibility.

  Response: Revised result section have addressed your comments.

Discussion:

- While the Discussion is generally well aligned with the results, some statements could be interpreted as implying causal relationships (e.g., references to “causal direction” or food environments “increasing BMI”). Given that all included studies are cross sectional, it would be helpful to more consistently qualify interpretations and avoid language that may overstate causal inference.

  Response: Terminologies like Causal Direction have been referenced from other findings. We have removed few of the languages that overstate causal inference.

- The Discussion occasionally places strong confidence in findings from individual studies described as having “high methodological rigor.” Given the limited number of included studies, their heterogeneity, and the cross sectional design, moderating this language or more explicitly acknowledging residual uncertainty would strengthen the interpretation.

  Response: Thank you.

- Line 323: The term “ultra-processed foods (UPFs)” is first introduced here and discussed repeatedly in the Discussion, yet it is not defined, operationalized, or referenced in the review’s objectives, search strategy, eligibility criteria, or data extraction methods. As currently written, UPFs appear to be introduced post hoc and treated interchangeably with “junk food,” without clarification that included studies actually assessed UPFs or used an established classification system (e.g., NOVA). This creates conceptual inconsistency between the Methods and Discussion. The authors should either clearly define and justify the integration of UPFs within the scope of the review, or limit interpretation to the exposure variables explicitly assessed in the included studies.

  Response: We have included NOVA definition for ultra-processed food and distinguish it from junk food with citation added [20]. We have also re-titled our manuscript to avoid any confusion among readers. Search strategy for core concepts is also further clarified.

Line: Line: 69 – 71 & 107 - 110

Thank you for going through this response letter. We hope this letter along with the revised manuscript will address all your valuable comments.

Sincerely,

Author of the manuscript
