# Supplementary material for: Association between unhealthy snack food and beverage consumption and the nutritional status of Nepalese children and adolescents: A systematic review
Source: PLoS One. 2026 Jun 25;21(6):e0352341. doi: 10.1371/journal.pone.0352341 (PMC13298750; doi:10.1371/journal.pone.0352341)
Supplement: Attachment — Submitted filename: Response_to_Reviewers_auresp_2.pdf [file pone.0352341.s006.pdf]

Dear Editor,
PLOS ONE
[PONE-D-25-64936R1]

We author thank you for your continuous precious time and effort to help us improvise the manuscript with your second round of valuable comments and concern. We believe this will further enhance and strengthen the manuscript. We author found that Editor mostly pointed out **five technical concerns** and simultaneously **three structural suggestions**. Those eight strong concerns & suggestions are now addressed with the changes in the manuscript.

Kindly go through the responses below.

Comments and Suggestions	Response by the author	Changes Visible
**Technical Issue 1.** Clarification of review registration.	We thank the Editor for highlighting this important point regarding methodological transparency. We acknowledge that a formal review protocol was not prospectively registered in an international database such as PROSPERO. Although we replied to reviewer concern on this earlier, we missed to mention it clearly in our manuscript.<br><br>To ensure transparency and minimize reporting bias, we strictly adhered to the standard Preferred Reporting Items for Systematic Reviews and Meta-Analyses (PRISMA) guidelines throughout the design, execution, and reporting of this study. We have explicitly clarified the lack of prospective registration in the Methods section under the **"Study design (2.1)"** subheading	Line: 81 - 83
**Technical Issue 2 and 3.** Clarification of inclusion criteria and exposure definitions.	We completely agree with the Editor that the terminology surrounding "unhealthy snack food and beverage consumption" required more rigorous operationalization.	Line: 88 - 108

	To resolve this ambiguity and ensure conceptual consistency, we have completely revised the **Study Selection Criteria (2.2)** section in the Methods. We have explicitly specified that our exposure variables encompass the various operational definitions found across Nepalese literature, including **junk foods, ultra-processed foods (UPFs), packaged snacks, sugar-sweetened beverages (SSBs), and fast foods.** We have also reformatted these criteria into clear bullet points to significantly improve reporting clarity and organization.	
**Technical Issue 4.**<br>Handling of urban–rural differences.	We recognize that the dietary environments of urban, peri-urban, and rural Nepal vary substantially.<br><br>To address this, we had explicitly stratified our results table by setting (Urban, Peri-urban, Rural, or Mixed) to allow for easier visual comparison.<br><br>**We have also now clearly stated on what basis the study areas were categorized at Data extraction (2.6).**<br><br>**Furthermore, we have now expanded our Discussion section to critically evaluate how this urban-heavy distribution (n=6 urban, n=3 peri-urban vs. n=1 rural) affects the generalizability of our findings and highlighted as a literature gap.**	Line: 160 – 165<br><br>Line: 300 - 308
**Technical Issue 5.**<br>Causal Interpretation	We have now fully sanitized this across the Title, Abstract, Introduction, Results (Table 3), Discussion, and Conclusion.	Title updated<br><br>Abstract: Line 1-4, 7-9, 14-15, & 22-25.

		Introduction: Line 36, 41-42, 46-49, & 75-77<br><br>Result Table 3: 211-214<br><br>Discussion: Line 249-254, 263-267, 276-277 & 294-297<br><br>Conclusion: Line 310-312 & 317-321
**Structural Suggestion 1.** Improve the Organization	We have fixed sentence flows, structured text blocks, and resolved contradictions simultaneously addressing Technical Issue 5 above.	
**Structural Suggestion 2.** Clearer Reporting	This revision has standardized statistical phrasings and epidemiological language. In addition, we have re-framed conclusion as **Conclusion and Recommendations**. By incorporating the recommendations, we aim to fulfill the editor's suggestion for improved organization and clearer reporting.<br><br>Thanks to the PLOS ONE submission guideline (Submission Guidelines | PLOS One) that explicitly lists a standalone "Conclusions" section as optional, giving full creative control to us on how to wrap up our discussion.	Line: 326-335
**Structural Suggestion 3.** Stronger Contextual Discussion	Tied the findings explicitly to Nepal's unique geographical, structural, and federalized landscape through the discussion.	

We would like to again sincerely thank the Academic Editor for pointing out these critical areas regarding methodological transparency and conceptual consistency. In the previous round of revisions, our primary focus was dedicated to addressing the extensive and detailed feedback provided by the four peer reviewers. As a result, some of these broader structural refinements were not fully realized. In this second revision, we have comprehensively addressed all five technical concerns and implemented your three structural suggestions, which we agree have substantially enhanced the precision and clarity of our manuscript.

Sincerely,

Author of the manuscript
