# Supplementary material for: Association between unhealthy snack food and beverage consumption and the nutritional status of Nepalese children and adolescents: A systematic review
Source: PLoS One. 2026 Jun 25;21(6):e0352341. doi: 10.1371/journal.pone.0352341 (PMC13298750; doi:10.1371/journal.pone.0352341)
Supplement: Peer review history

# Author comment: Author response to Decision Letter 0

**DOI:** 10.1371/journal.pone.0352341.r001

4 Dec 2025

Attachment Submitted filename: Response-letter-Updated.pdf

# Peer review: Decision Letter 0

**DOI:** 10.1371/journal.pone.0352341.r002

**Authors:** Bijit Biswas

15 Apr 2026

PONE-D-25-64936

The association between consumption of junk food and nutritional status among children and adolescents in Nepal: A Systematic Review.

PLOS One

Dear Dr. Parajuli,

Thank you for submitting your manuscript to PLOS ONE. After careful consideration, we feel that it has merit but does not fully meet PLOS ONE’s publication criteria as it currently stands. Therefore, we invite you to submit a revised version of the manuscript that addresses the points raised during the review process.

==============================

**ACADEMIC EDITOR:**  Thank you for your submission. The topic is relevant and contextually important; however, the manuscript requires major revision before it can be considered further. Key concerns include limitations in the search strategy and study selection (including exclusion of many full texts), insufficient methodological transparency (registration, eligibility criteria, and risk-of-bias assessment), and overinterpretation of findings derived from cross-sectional studies. The manuscript would benefit from improved structure and clarity, clearer definitions of exposure (junk food vs. ultra-processed foods), strengthened data synthesis (including stratification where feasible), and more specific, contextually grounded discussion and policy implications. Please address all reviewer comments comprehensively.

==============================

Please submit your revised manuscript by May 30 2026 11:59PM. If you will need more time than this to complete your revisions, please reply to this message or contact the journal office at plosone@plos.org. When you're ready to submit your revision, log on to https://www.editorialmanager.com/pone/ and select the 'Submissions Needing Revision' folder to locate your manuscript file.

Please include the following items when submitting your revised manuscript:

- A letter that responds to each point raised by the academic editor and reviewer(s). You should upload this letter as a separate file labeled 'Response to Reviewers'.
- A marked-up copy of your manuscript that highlights changes made to the original version. You should upload this as a separate file labeled 'Revised Manuscript with Track Changes'.
- An unmarked version of your revised paper without tracked changes. You should upload this as a separate file labeled 'Manuscript'.

If you would like to make changes to your financial disclosure, please include your updated statement in your cover letter. Guidelines for resubmitting your figure files are available below the reviewer comments at the end of this letter.

If applicable, we recommend that you deposit your laboratory protocols in protocols.io to enhance the reproducibility of your results. Protocols.io assigns your protocol its own identifier (DOI) so that it can be cited independently in the future. For instructions see: https://journals.plos.org/plosone/s/submission-guidelines#loc-laboratory-protocols. Additionally, PLOS ONE offers an option for publishing peer-reviewed Lab Protocol articles, which describe protocols hosted on protocols.io. Read more information on sharing protocols at https://plos.org/protocols?utm_medium=editorial-email&utm_source=authorletters&utm_campaign=protocols.

As the corresponding author, your ORCID iD is verified in the submission system and will appear in the published article. PLOS supports the use of ORCID, and we encourage all coauthors to register for an ORCID iD and use it as well. Please encourage your coauthors to verify their ORCID iD within the submission system before final acceptance, as unverified ORCID iDs will not appear in the published article. *Only*  the individual author can complete the verification step; PLOS staff *cannot*  verify ORCID iDs on behalf of authors.

We look forward to receiving your revised manuscript.

Kind regards,

Bijit Biswas, MBBS, MD, DNB

Academic Editor

PLOS One

Journal Requirements:

When submitting your revision, we need you to address these additional requirements.

1. Please ensure that your manuscript meets PLOS ONE's style requirements, including those for file naming. The PLOS ONE style templates can be found at

https://journals.plos.org/plosone/s/file?id=wjVg/PLOSOne_formatting_sample_main_body.pdf and https://journals.plos.org/plosone/s/file?id=ba62/PLOSOne_formatting_sample_title_authors_affiliations.pdf

If the reviewer comments include a recommendation to cite specific previously published works, please review and evaluate these publications to determine whether they are relevant and should be cited. There is no requirement to cite these works unless the editor has indicated otherwise.

Additional Editor Comments:

Thank you for your submission. The topic is relevant and contextually important; however, the manuscript requires major revision before it can be considered further. Key concerns include limitations in the search strategy and study selection (including exclusion of many full texts), insufficient methodological transparency (registration, eligibility criteria, and risk-of-bias assessment), and overinterpretation of findings derived from cross-sectional studies. The manuscript would benefit from improved structure and clarity, clearer definitions of exposure (junk food vs. ultra-processed foods), strengthened data synthesis (including stratification where feasible), and more specific, contextually grounded discussion and policy implications. Please address all reviewer comments comprehensively.

[Note: HTML markup is below. Please do not edit.]

Reviewers' comments:

Reviewer's Responses to Questions

**Comments to the Author**

1. Is the manuscript technically sound, and do the data support the conclusions?

The manuscript must describe a technically sound piece of scientific research with data that supports the conclusions. Experiments must have been conducted rigorously, with appropriate controls, replication, and sample sizes. The conclusions must be drawn appropriately based on the data presented.

Reviewer #1: No

Reviewer #2: Yes

Reviewer #3: Partly

Reviewer #4: Partly

**********

2. Has the statistical analysis been performed appropriately and rigorously?

Reviewer #1: N/A

Reviewer #2: Yes

Reviewer #3: N/A

Reviewer #4: N/A

**********

3. Have the authors made all data underlying the findings in their manuscript fully available?

The PLOS Data policy requires authors to make all data underlying the findings described in their manuscript fully available without restriction, with rare exception (please refer to the Data Availability Statement in the manuscript PDF file). The data should be provided as part of the manuscript or its supporting information, or deposited to a public repository. For example, in addition to summary statistics, the data points behind means, medians and variance measures should be available. If there are restrictions on publicly sharing data—e.g. participant privacy or use of data from a third party—those must be specified.

Reviewer #1: Yes

Reviewer #2: Yes

Reviewer #3: Yes

Reviewer #4: Yes

**********

4. Is the manuscript presented in an intelligible fashion and written in standard English?

PLOS ONE does not copyedit accepted manuscripts, so the language in submitted articles must be clear, correct, and unambiguous. Any typographical or grammatical errors should be corrected at revision, so please note any specific errors here.

Reviewer #1: No

Reviewer #2: Yes

Reviewer #3: Yes

Reviewer #4: Yes

**********

5. Review Comments to the Author

Please use the space provided to explain your answers to the questions above. You may also include additional comments for the author, including concerns about dual publication, research ethics, or publication ethics. (Please upload your review as an attachment if it exceeds 20,000 characters)

Reviewer #1: Dear Author

While the study is relevant to public health, especially for low- and middle-income countries, and includes several elements of the PRISMA framework, including a PRISMA flow diagram, it presents significant methodological limitations that affect its robustness and reproducibility, thus compromising its scientific quality.

One limitation is the time frame restriction (2019–2025), which is relatively short for a systematic review and could introduce selection bias by excluding relevant evidence published in earlier periods. Furthermore, 150 articles were excluded due to lack of access to the full text. This considerable number represents a significant methodological weakness, as obtaining the full texts of potentially eligible studies is an expected part of the process in this type of research.

It is also observed that the studies included in the review come exclusively from a single country, which considerably limits the generalizability and international scope of the findings. For a high-impact journal, systematic reviews are expected to integrate evidence from multiple geographical contexts to strengthen the external validity and global relevance of the results. In its current form, the exclusively national nature of the evidence restricts the review's contribution and reduces its suitability for journals at this indexing level.

Furthermore, there are other formal aspects that need improvement. The abstract does not specify the search period or the inclusion criteria used. The introduction needs improvement in its logical sequence; it is disorganized, repeats ideas in different paragraphs, and does not delve deeply into aspects that could be useful in the discussion, thus hindering comprehension. The methodology section does not detail the scoring system used to categorize studies as low, moderate, or high risk; it only states that the JBI was used.

The results show a tendency to suggest causal relationships, even though the included studies are cross-sectional in design, which limits the conclusions. While the authors acknowledge some limitations—such as the exclusive inclusion of cross-sectional studies, the limited time frame, and the small number of studies—these should be discussed in greater depth considering their impact on the interpretation of the findings. In this case, the main contribution is limited to the Nepalese context, which may be acceptable for regional journals but is insufficient for a high-impact journal.

Consequently, considering the methodological weaknesses noted, the limited coverage of the evidence, the exclusion of a large number of articles due to lack of access, the small final number of included studies (n=11), and the deficiencies in the manuscript's structure and writing, it is concluded that the article does not meet the methodological and scientific standards expected of a journal such as PLOS ONE. For these reasons, the recommendation is that the manuscript not be accepted for publication.

Reviewer #2: 1. The gender distribution of participants in each included study should be reported, as dietary habits may differ between males and females.

2. The specific types of junk food examined in each individual study must be clearly described. The current definition is overly broad, and detailed itemized food categories should be provided in the Results section.

3. Only 1 study was conducted exclusively in rural areas and 3 in peri‑urban areas, leading to an imbalanced sample distribution across urban, peri‑urban, and rural settings. Consequently, the conclusions regarding rural populations lack credibility. It is strongly recommended that the authors explicitly acknowledge this sampling bias in the Results section and note the limited generalizability of rural findings. Alternatively, high‑quality rural studies should be added.

4. Figure 2, which presents the range of nutritional status percentages, is of low quality because it lacks error bars or confidence intervals. The figure should be revised to a bar chart stratified by urban, peri‑urban, and rural areas.

5. When synthesizing data, the authors should analyze the association between the frequency of junk food consumption and the risk of malnutrition (e.g., dose–response relationships).

6. The topic of junk food intake and nutritional status in children is relatively conventional; however, the focus on the Nepalese population provides some novelty. The authors must strengthen the discussion of local contextual factors in Nepal—including the earthquake, urbanization, food policies, and economic development—to more thoroughly explain urban–rural disparities. Without this, the novelty of the study cannot be sufficiently highlighted.

7. In the Discussion section, the authors should address the biological and social mechanisms linking junk food to overweight/obesity and malnutrition in rural areas, such as high sugar/fat content, monotonous diets, and caregivers’ nutritional knowledge.

8. The current policy recommendations are too general. Specific, actionable, and context‑appropriate measures should be proposed to improve practical utility.

Reviewer #3: Thank you for inviting me to review the manuscript titled “The association between consumption of junk food and nutritional status among

children and adolescents in Nepal: A Systematic Review.” This study uses a systematic review to summarize the relationship between the nutritional status of children and adolescents in Nepal and junk food consumption. After a careful review, I have several questions I would like the authors to address:

1. Was this systematic review registered?

2. What is the current nutritional status of children and adolescents in Nepal? What are the common influencing factors? If there is a clear polarization, is it appropriate to focus solely on junk food?

3. Why were children and adolescents not analyzed separately? How did the authors account for puberty, which is a very important variable?

4. Is access to junk food the same for all children and adolescents? Furthermore, how do the prices of junk food compare to those of healthy foods or home-cooked meals? My concern here is whether economic factors might be driving children toward junk food consumption.

Reviewer #4: This manuscript addresses an important and under‑studied public health issue by synthesizing evidence on junk‑food consumption and nutritional status among children and adolescents in Nepal. The focus on geographic context (urban, peri‑urban, and rural settings) is a valuable contribution and offers policy‑relevant insights for settings undergoing nutrition transition. With revisions to improve methodological clarity, conceptual consistency, and precision of reporting, this review has the potential to make a meaningful addition to the literature.

Abstract:

The abstract states that the review aims to assess the relationship between children’s nutritional status in Nepal and junk food consumption. However, the Results portion of the abstract focuses primarily on geographic distribution (urban, peri‑urban, rural) rather than summarizing findings related to nutritional status outcomes. Consider revising the abstract to better reflect the stated aim by highlighting key nutritional status findings.

Introduction:

The overall structure of the introduction would benefit from greater consistency (e.g., presenting global context first, followed by regional and then Nepal specific data for each major theme).

While the description of BMI and its association with overweight/obesity is factually correct, BMI alone is an imperfect proxy for nutritional status. A brief acknowledgment of its limitations (e.g., inability to distinguish body composition) would provide a more balanced framing.

Beginning on line 57: Several prevalence statistics lack clear geographic context. For example:

“In 2024, an estimated 35 million children under the age of 5 years were overweight and obese” — please specify whether this refers to global, regional, or country level estimates.

Similar clarification is needed for statistics referring to Asia and for the figure stating that “more than 390 million children and adolescents aged 5–19 were overweight.”

Line 58: "The same inclining trend..." is introduced without a prior description of a trend; this should be revised for logical continuity.

Line 60: "In 2024, Asia accounted for about half of all children under five 61 who were overweight or obese" could be read as referring either to the global distribution of overweight/obese children, or to the prevalence within Asia itself. Clarifying the intended interpretation would improve clarity.

Line 75: The statement noting that 70% of obese adolescents and 30% of overweight children become obese adults is compelling and would be more effective if introduced earlier to motivate the public health relevance before presenting prevalence statistics.

The discussion of undernutrition focuses largely on short term consequences (e.g., mortality under age five). Consider briefly addressing long term consequences (health, economic, etc.) of childhood undernutrition to further strengthen the argument of why this is important.

Line 88: The shift from under and overnutrition to eating patterns and junk food consumption is abrupt. The introduction would benefit from a clearer conceptual bridge explicitly linking dietary patterns (including junk/ultra processed foods) to both under and overnutrition.

Line 104: The paragraph beginning here is considerably more detailed than necessary. Consider front loading the key takeaway (i.e., dietary shifts toward packaged convenience foods in Nepal) and then briefly summarizing supporting evidence, rather than presenting detailed study descriptions.

Methods:

Section 2.1: This section repeats methodological details that are described more fully in subsequent subsections (e.g., search strategy, eligibility criteria, screening, data extraction). It could be streamlined to briefly state the review design and PRISMA adherence, with operational details retained in Sections 2.2–2.6.

Section 2.2: The exclusion criteria largely mirror the inverse of the inclusion criteria, which limits their usefulness for understanding screening decisions. Clarifying explicit exclusion rules (e.g., mixed age samples without separable child data, multi country studies without Nepal specific results, non–peer reviewed publications) would strengthen methodological transparency.

Section 2.4: Please provide a citation for the reference management software (Zotero).

Section 2.6: The description of extracted variables could be streamlined to emphasize analytically meaningful elements. Leading with bibliographic information adds little methodological clarity, as these details are implicit in any systematic review. Additionally, given that only cross sectional studies were eligible, it is unclear what analytic purpose extracting “study design” served. Clarification or revision to focus on variables informing synthesis (e.g., exposure and outcome measurement, confounding, statistical approaches) is suggested.

Results:

Section 3.1: The step-by-step narrative of record counts duplicates information shown in the PRISMA flow diagram. For clarity and concision, the authors may wish to let Figure 1 present the detailed screening numbers and retain only a brief summary of the final inclusion in the text.

Table 1: Table 1 is helpful, but the rationale for the overall risk-of-bias ratings would benefit from greater transparency and consistency. The brief narrative comments vary in focus and do not clearly indicate which JBI checklist domains most strongly influenced each appraisal. I suggest standardizing comments to explicitly reference key checklist items (e.g., exposure measurement, confounding control, sampling, outcome assessment) or briefly noting the primary domains driving the Low/Moderate/High rating. This would improve interpretability and comparability across included studies.

Lines 240-245 and Table 2: The description of the narrative synthesis and Table 2 is awkwardly worded and somewhat unclear. Phrases such as “summarized and analysed narratively by presenting them in the below table” are redundant and imprecise, and the listed table domains mix bibliographic details, methods, and findings without a clear organizing rationale. In addition, the justification for not conducting statistical analyses is not strictly necessary given the absence of meta-analysis. Streamlining this section to clearly describe the contents and purpose of Table 2, and briefly justify the use of narrative synthesis, would improve readability and methodological clarity.

Figure 2: The table title refers to “Children,” while the caption refers to “Children and Adolescents.” Please clarify which population is represented and specify the age range. If feasible based on available data, stratifying by age group may strengthen this figure.

Lines 277–287: This paragraph contains several interpretive claims without explicit citations. Even if these statements reflect synthesis by the authors, referencing the specific included studies that support each point would strengthen credibility.

Discussion:

While the Discussion is generally well aligned with the results, some statements could be interpreted as implying causal relationships (e.g., references to “causal direction” or food environments “increasing BMI”). Given that all included studies are cross sectional, it would be helpful to more consistently qualify interpretations and avoid language that may overstate causal inference.

The Discussion occasionally places strong confidence in findings from individual studies described as having “high methodological rigor.” Given the limited number of included studies, their heterogeneity, and the cross sectional design, moderating this language or more explicitly acknowledging residual uncertainty would strengthen the interpretation.

Line 323: The term “ultra‑processed foods (UPFs)” is first introduced here and discussed repeatedly in the Discussion, yet it is not defined, operationalized, or referenced in the review’s objectives, search strategy, eligibility criteria, or data extraction methods. As currently written, UPFs appear to be introduced post hoc and treated interchangeably with “junk food,” without clarification that included studies actually assessed UPFs or used an established classification system (e.g., NOVA). This creates conceptual inconsistency between the Methods and Discussion. The authors should either clearly define and justify the integration of UPFs within the scope of the review, or limit interpretation to the exposure variables explicitly assessed in the included studies.

General comments:

The manuscript contains multiple instances of subject verb agreement errors (singular vs. plural). A careful grammatical review of the entire manuscript is recommended.

Consider acknowledging the lack of a universally agreed upon definition of ultra processed foods, as this has implications for exposure measurement across included studies.

**********

6. PLOS authors have the option to publish the peer review history of their article (what does this mean?). If published, this will include your full peer review and any attached files.

If you choose “no”, your identity will remain anonymous but your review may still be made public.

**Do you want your identity to be public for this peer review?**  For information about this choice, including consent withdrawal, please see our Privacy Policy.

Reviewer #1: No

Reviewer #2: No

Reviewer #3: **Yes:**  Yin Li

Reviewer #4: No

**********

[NOTE: If reviewer comments were submitted as an attachment file, they will be attached to this email and accessible via the submission site. Please log into your account, locate the manuscript record, and check for the action link "View Attachments". If this link does not appear, there are no attachment files.]

To ensure your figures meet our technical requirements, please review our figure guidelines: https://journals.plos.org/plosone/s/figures

You may also use PLOS’s free figure tool, NAAS, to help you prepare publication quality figures: https://journals.plos.org/plosone/s/figures#loc-tools-for-figure-preparation.

NAAS will assess whether your figures meet our technical requirements by comparing each figure against our figure specifications.

# Author comment: Author response to Decision Letter 1

**DOI:** 10.1371/journal.pone.0352341.r003

1 May 2026

Dear Editor and all 4 Reviewers,

Thank you for your precious time and effort to help us improvise the manuscript with your valuable comments and concern. We truly acknowledge your contribution. We have submitted all the responses to comments and concerns made by Editor and reviewers in one letter as "Response to Reviewers". We hope this letter along with the revised manuscript will address all your valuable comments.

Sincerely,

Attachment Submitted filename: Response to Reviewers.pdf

# Editor report: Decision Letter 1

**DOI:** 10.1371/journal.pone.0352341.r004

**Authors:** Bijit Biswas

24 May 2026

PONE-D-25-64936R1

Impact of Unhealthy Snack Food and Beverage Consumption on the Nutritional Status of Nepalese Children and Adolescents: A Systematic Review.

PLOS One

Dear Dr. Parajuli,

Thank you for submitting your manuscript to PLOS ONE. After careful consideration, we feel that it has merit but does not fully meet PLOS ONE’s publication criteria as it currently stands. Therefore, we invite you to submit a revised version of the manuscript that addresses the points raised during the review process.

==============================

**ACADEMIC EDITOR:**  The manuscript addresses an important public health topic with relevance to Nepal and other LMIC settings. However, major revisions are needed to strengthen methodological transparency, conceptual consistency, and interpretation of findings. Key concerns include clarification of review registration, inclusion criteria, exposure definitions, handling of urban–rural differences, and avoidance of causal interpretations from cross-sectional evidence. Improved organization, clearer reporting, and stronger contextual discussion would substantially enhance the manuscript.

==============================

Please submit your revised manuscript by Jul 08 2026 11:59PM. If you will need more time than this to complete your revisions, please reply to this message or contact the journal office at plosone@plos.org. When you're ready to submit your revision, log on to https://www.editorialmanager.com/pone/ and select the 'Submissions Needing Revision' folder to locate your manuscript file.

Please include the following items when submitting your revised manuscript:

- A letter that responds to each point raised by the academic editor and reviewer(s). You should upload this letter as a separate file labeled 'Response to Reviewers'.
- A marked-up copy of your manuscript that highlights changes made to the original version. You should upload this as a separate file labeled 'Revised Manuscript with Track Changes'.
- An unmarked version of your revised paper without tracked changes. You should upload this as a separate file labeled 'Manuscript'.

If you would like to make changes to your financial disclosure, please include your updated statement in your cover letter. Guidelines for resubmitting your figure files are available below the reviewer comments at the end of this letter.

If applicable, we recommend that you deposit your laboratory protocols in protocols.io to enhance the reproducibility of your results. Protocols.io assigns your protocol its own identifier (DOI) so that it can be cited independently in the future. For instructions see: https://journals.plos.org/plosone/s/submission-guidelines#loc-laboratory-protocols. Additionally, PLOS ONE offers an option for publishing peer-reviewed Lab Protocol articles, which describe protocols hosted on protocols.io. Read more information on sharing protocols at https://plos.org/protocols?utm_medium=editorial-email&utm_source=authorletters&utm_campaign=protocols.

As the corresponding author, your ORCID iD is verified in the submission system and will appear in the published article. PLOS supports the use of ORCID, and we encourage all coauthors to register for an ORCID iD and use it as well. Please encourage your coauthors to verify their ORCID iD within the submission system before final acceptance, as unverified ORCID iDs will not appear in the published article. *Only*  the individual author can complete the verification step; PLOS staff *cannot*  verify ORCID iDs on behalf of authors.

We look forward to receiving your revised manuscript.

Kind regards,

Bijit Biswas, MBBS, MD, DNB

Academic Editor

PLOS One

**Journal Requirements:**

If the reviewer comments include a recommendation to cite specific previously published works, please review and evaluate these publications to determine whether they are relevant and should be cited. There is no requirement to cite these works unless the editor has indicated otherwise.

**Additional Editor Comments:**

The manuscript addresses an important public health topic with relevance to Nepal and other LMIC settings. However, major revisions are needed to strengthen methodological transparency, conceptual consistency, and interpretation of findings. Key concerns include clarification of review registration, inclusion criteria, exposure definitions, handling of urban–rural differences, and avoidance of causal interpretations from cross-sectional evidence. Improved organization, clearer reporting, and stronger contextual discussion would substantially enhance the manuscript.

[Note: HTML markup is below. Please do not edit.]

[NOTE: If reviewer comments were submitted as an attachment file, they will be attached to this email and accessible via the submission site. Please log into your account, locate the manuscript record, and check for the action link "View Attachments". If this link does not appear, there are no attachment files.]

To ensure your figures meet our technical requirements, please review our figure guidelines: https://journals.plos.org/plosone/s/figures

You may also use PLOS’s free figure tool, NAAS, to help you prepare publication quality figures: https://journals.plos.org/plosone/s/figures#loc-tools-for-figure-preparation.

NAAS will assess whether your figures meet our technical requirements by comparing each figure against our figure specifications.

# Author comment: Author response to Decision Letter 2

**DOI:** 10.1371/journal.pone.0352341.r005

25 May 2026

Dear Academic Editor,

We have thoroughly gone through this second round of revision. Unlike earlier complete and deep revision including data work and methodological work, this second revision was more towards overall enhancement of the manuscript to meet the standard of PLOS ONE publication. We believe now our manuscript is meeting the PLOS ONE publication criteria. Any remaining typo error or minor error can be corrected during proof reading. Thank you.

Attachment Submitted filename: Response_to_Reviewers_auresp_2.pdf

# Editor report: Decision Letter 2

**DOI:** 10.1371/journal.pone.0352341.r006

**Authors:** Bijit Biswas

10 Jun 2026

Association Between Unhealthy Snack Food and Beverage Consumption and the Nutritional Status of Nepalese Children and Adolescents: A Systematic Review.

PONE-D-25-64936R2

Dear Dr. Rameshwor Parajuli,

We’re pleased to inform you that your manuscript has been judged scientifically suitable for publication and will be formally accepted for publication once it meets all outstanding technical requirements.

Within one week, you’ll receive an e-mail detailing the required amendments. When these have been addressed, you’ll receive a formal acceptance letter and your manuscript will be scheduled for publication.

An invoice will be generated when your article is formally accepted. Please note, if your institution has a publishing partnership with PLOS and your article meets the relevant criteria, all or part of your publication costs will be covered. Please make sure your user information is up-to-date by logging into Editorial Manager at Editorial Manager® and clicking the ‘Update My Information' link at the top of the page. For questions related to billing, please contact billing support.

If your institution or institutions have a press office, please notify them about your upcoming paper to help maximize its impact. If they’ll be preparing press materials, please inform our press team as soon as possible -- no later than 48 hours after receiving the formal acceptance. Your manuscript will remain under strict press embargo until 2 pm Eastern Time on the date of publication. For more information, please contact onepress@plos.org.

Kind regards,

Bijit Biswas, MBBS, MD, DNB

Academic Editor

PLOS One

Additional Editor Comments (optional):

Thank you for carefully revising the manuscript and for providing detailed responses to the reviewers' comments. The revised version demonstrates substantial improvements in methodological transparency, conceptual clarity, organization, and contextual interpretation. The authors have appropriately clarified key aspects of the review and have strengthened the discussion and reporting. I have no further major concerns and consider the manuscript suitable for publication.

# Editor report: Acceptance letter

**DOI:** 10.1371/journal.pone.0352341.r007

**Authors:** Bijit Biswas

PONE-D-25-64936R2

PLOS One

Dear Dr. Parajuli,

I'm pleased to inform you that your manuscript has been deemed suitable for publication in PLOS One. Congratulations! Your manuscript is now being handed over to our production team.

At this stage, our production department will prepare your paper for publication. This includes ensuring the following:

* All references, tables, and figures are properly cited

* All relevant supporting information is included in the manuscript submission,

* There are no issues that prevent the paper from being properly typeset

You will receive further instructions from the production team, including instructions on how to review your proof when it is ready. Please keep in mind that we are working through a large volume of accepted articles, so please give us a few days to review your paper and let you know the next and final steps.

Lastly, if your institution or institutions have a press office, please let them know about your upcoming paper now to help maximize its impact. If they'll be preparing press materials, please inform our press team within the next 48 hours. Your manuscript will remain under strict press embargo until 2 pm Eastern Time on the date of publication. For more information, please contact onepress@plos.org.

You will receive an invoice from PLOS for your publication fee after your manuscript has reached the completed accept phase. If you receive an email requesting payment before acceptance or for any other service, this may be a phishing scheme. Learn how to identify phishing emails and protect your accounts at https://explore.plos.org/phishing.

If we can help with anything else, please email us at customercare@plos.org.

Thank you for submitting your work to PLOS ONE and supporting open access.

Kind regards,

PLOS ONE Editorial Office Staff

on behalf of

Dr. Bijit Biswas

Academic Editor

PLOS One
